# Supergroup algorithm and knowledge graph construction in museum digital display platform

**DOI:** 10.1016/j.heliyon.2024.e38076

**Published:** 2024-09-23

**Authors:** Liping Su, Hongli Liu, Wenru Zhao

**Affiliations:** a College of Fine Arts and Design, Hebei Normal University for Nationalities, Chengde, 067000, Hebei, China; b Graduate School, University of Perpetual Help System Dalta, Manila, 0900, Philippines

**Keywords:** Knowledge graph, Museum digital display platform, Supergroup algorithm, K-means algorithm, Cultural relic knowledge extraction

## Abstract

In response to the problems of low entity recognition accuracy, low user satisfaction, and weak interactivity in the construction of knowledge graph for digital display of museum cultural relics, this article studied the application of supergroup algorithms and knowledge graph construction in museum digital display platforms to solve the existing problems. By utilizing the K-means algorithm in the supergroup algorithm to conduct a survey of visitors to Museum A and analyze the behavior of 180 selected visitors, the display effect and audience satisfaction can be improved. Various knowledge graph technologies were utilized to construct a knowledge graph of museum cultural relics. Various knowledge resources in museums were associated and integrated, and through the collection and processing of museum cultural relic data, cultural relic ontology construction and relationship extraction were achieved, providing viewers with richer and more in-depth display content. Through experiments, it was found that the visitor satisfaction rate based on the K-means algorithm was above 92.68 %, and the average visitor satisfaction rate after 10 experiments was 94.25 %. The accuracy, recall, and F1 values of the museum cultural relics knowledge graph studied in this article were 90.12 %, 84.69 %, and 82.23 %, respectively, which were much higher than other types of knowledge graphs. By applying these advanced technologies to the digital display platform of museums, not only can the visitor experience be improved, but also the digitalization process of museums can be promoted, contributing to cultural dissemination and development.

## 1 Introduction

With the continuous improvement of museum informatization and intelligence, the role of museums in knowledge acquisition is constantly deepening, especially their cooperation in knowledge sharing and dissemination is deepening. The demand for knowledge acquisition from a large number of museum visitors and users is also constantly increasing. However, due to the fact that museums themselves are a knowledge base with a long history of development, the acquisition and utilization of their knowledge information have not been well adapted to the development of the digital network era, and many historical knowledge and cultural relic information have not been better excavated. In this context, supergroup algorithms and knowledge graph construction have become important technologies supporting innovation in museum digital display platforms. In the digital exhibition platform of museums, excellent algorithms can intelligently promote exhibits that meet the needs of visitors based on their personal characteristics such as interests, preferences, and behaviors. The construction of a knowledge graph is to express various entities and their relationships in the form of a graph, thereby creating a good knowledge connection network, allowing audiences to have a more personalized and in-depth visiting experience, and better fulfilling the positive role of museums in promoting cultural history.

The following elements primarily represent the research's new contribution to the field of museum digitization. 1) Introduction of the supergroup algorithm: the analysis and processing of data related to museum cultural relics is done in this article using the supergroup algorithm. This method can produce finer data support and mine possible correlations between cultural relics more accurately. It is discovered through comparison experiments that the supergroup algorithm performs significantly better than conventional algorithms in numerous areas. 2) Optimization of knowledge graph creation: this research enhances the accuracy and use of the knowledge graph by taking into account both the demands of the audience to visit and the features of museum cultural treasures.

The museum digital display platform is a platform that uses digital technology to display museum cultural relics and information. This platform can convert physical exhibits into virtual exhibits through digital means, achieve online discussions and interactions, and allow visitors to understand the background and connotation of cultural relics in real-time without being limited by time and location [1,2]. Gran Anne-Britt introduced three datasets, which mainly collected data on museum cultural relics. Using three indicators of user background, behavior, and values, the three datasets were identified to describe who used the digital platform and what specific user preferences were [3]. Zidianakis Emmanouil introduced the Invisible Museum platform, which is a user centered platform that allows users to create interactive and immersive virtual exhibitions using a unified creative environment. The design of the platform itself follows a people-oriented approach, helping museum visitors to better view exhibits [4]. Based on the co-production of stories, Liu Peng understood the interrelationships between museum visitors, museum spaces, and cultural heritage stories, and explored the quality of cultural museum experiences [5]. Micoli Laura Loredana designed an interactive museum collection application where there are multiple clear logical connections between cultural relics, referring to elements of intangible cultural heritage [6]. Khundam Chaowanan focused on virtual museums and proposed a device independent interactive content design platform. The platform framework provides high-level abstractions of stories and interactions [7]. In summary, in-depth research has been conducted on museum digital display platforms, and some research results have been achieved. However, these studies still have shortcomings in entity extraction recognition of cultural relics and satisfaction rate of cultural relic recommendation.

The construction of museum knowledge graph is to use graph technology to integrate museum knowledge resources, aiming to display the related information of museum cultural relics and provide audiences with a more comprehensive and in-depth knowledge experience [8,9]. Li Yonghui conducted research on knowledge organization and service of Southern Dynasty tomb stone carving information resources through ontology based knowledge graph construction, which effectively improved the audience's immersion and interactivity, promoted the dissemination and utilization of cultural heritage, and provided a new perspective for the inheritance and protection of stone carving resources [10]. Poulovassilis Alexandra aimed to establish a comprehensive database of British museums, which required the design of a new knowledge graph to store data and metadata related to British museums for humanities scholars to browse and search museum data [11]. Wang Xiaoguang believed that using knowledge graphs can achieve the representation and association of information resources, and constructed an ontology model to standardize the entities, attributes, and relationships of Dunhuang knowledge. He used various techniques to process the obtained data for easy presentation in knowledge graph construction, effectively promoting the research, teaching, and dissemination of Dunhuang cultural heritage [12]. Chen Qingbei constructed a question and answer system based on the knowledge graph of Xizang's cultural relics, which can better protect and inherit Xizang's cultural heritage, use reptile technology to obtain Xizang's cultural relics data, and store it in the database [13]. Hou Wenjun proposed a graph representation learning model that integrates cultural attributes based on knowledge graph data from the mining and utilization of cultural resources in the ancient capital of Beijing. He constructed a domain specific feature matrix in the form of word vector concatenation. The experimental results showed that the learning model performed better [14]. Research has shown that constructing a knowledge graph of museum cultural relics can improve the efficiency of museum cultural relic dissemination and better extract physical data of cultural relics.

Im Bokhee proposed a pure supergroup algorithm version of the superalgebraic tensor product, which also applied to groups or their non-associative analogues-quasigroups and loops, unifying the structure of eight-element quaternions and dihedral groups [15]. For general linear supergroups, Marko FrantiSek considered a natural isomorphism and calculated the effect of odd supergroups on generator images [16]. The use of knowledge graph as side information to generate recommendations has aroused considerable interest. This method can not only alleviate the above problems to obtain more accurate recommendations, but also provide explanations for recommended projects. For this reason, Guo Qingyu conducted a systematic study on the recommendation system based on knowledge graph [17]. Li Zhifei proposed a heterogeneous graph neural network framework based on attention mechanism. Specifically, the neighbor characteristics of an entity are first aggregated under each relationship path, and then the importance of different relationship paths is understood through the relationship characteristics. Finally, the weight values of each feature and learning based on the relational path are aggregated to generate an embedded representation [18].

In the digital exhibition platform of museums, supergroup algorithm and knowledge graph construction are two interrelated core technologies. Supergroup algorithms are usually used to filter out the most important, interesting, and useful content from a large amount of digital content, while knowledge graph design organizes these contents in an easy to understand and query way, forming a knowledge network that is easy to understand and query. Firstly, the supergroup algorithm can recommend exhibits related to the audience's interests and behavior patterns. By analyzing a wide range of user feedback and behavior data, it helps museums optimize exhibition layout and content arrangement. The construction of knowledge graphs plays an important role in the digital display platform of museums. It can organize various exhibits, exhibitions, historical events, and their relationships and attributes, allowing visitors to understand the relationships and characteristics of each organization by querying knowledge graphs, thereby better understanding and appreciating exhibits.

Through the introduction of superior algorithms and the optimization of knowledge graph generation techniques, this research has brought new discoveries and advances to the field of digital display in museums. New concepts and techniques for the categorization, grouping, and correlation analysis of cultural relics are offered by the employment of the supergroup algorithm in the data processing of museum artifacts. This approach not only increases data processing efficiency and accuracy, but also creates a strong basis for the creation of knowledge graphs and personalized recommendation systems in the future. Additionally, it offers a more varied and comprehensive application strategy for museums’ digital displays.

## 2 Users of museum display platform based on K-means algorithm

The museum digital display platform is a platform that utilizes digital technology to showcase museum cultural relics and information. This platform combines museum experience with digital technology, providing visitors with rich, interactive, and personalized visiting experiences [19,20]. Museum digital display platforms should provide educational and interesting content to meet the needs of visitors of different age groups. The numerous collections and information in museums are systematically integrated and classified, making it convenient for audiences to quickly find the content they are interested in. This platform utilizes supergroup algorithms and knowledge graph technology to provide personalized recommendations based on customer interests and preferences, enhancing the visitor experience. Supergroup algorithm is an unsupervised machine learning method used to group similar objects or data points together to form groups called “identities” or “clusters”. The elements in these groups or categories are to some extent similar, while the data elements in different groups or categories are different. The application of super algorithms on museum digital display platforms can explore the relationships between collections and provide visitors with optimal and accurate viewing services. The basic information obtained through studying visitors provides good support for the creation of digital museums. It is very important to use the K-means algorithm in supergroup algorithms to simulate user behavior, and identify user needs and motivations.

The effectiveness of knowledge graph construction really depends on the selected algorithm and its implementation to a great extent. As a common clustering method, K-means algorithm may be used in entity clustering or relational clustering in knowledge graph construction. However, insufficient algorithm selection or implementation may indeed lead to suboptimal results, which mainly comes from the following aspects.

1. Applicability of algorithms: Not all algorithms are suitable for specific knowledge graph construction tasks. For example, K-means algorithm is suitable for clustering spherical clusters, but if the data distribution is complex or the shape of clusters is irregular, K-means may not be the best choice.
2. Parameter adjustment: Many algorithms have a series of parameters to be adjusted, such as the number of clusters k in K-means algorithm. If the parameters are not set properly, the performance of the algorithm may be affected.

### 2.1 Data Preparation stage

The cultural relics in museums are the best and most direct way for the public to understand, respect, and remember history [21,22]. When visiting a museum, visitors not only need to understand which type of cultural relics they prefer, but also need to understand the relationships between cultural relics. Therefore, this article conducts research on Museum A, which has completed the digital display of cultural relics. The collection of cultural relics is very large, and the types of cultural relics are also very diverse. Through these cultural relics, the social atmosphere and economic development situation at that time can be understood. The information of these cultural relics is mainly collected by the museum's data center, which includes the textual introduction and image information of the cultural relics. The textual information includes the name, excavation time, excavation location, type, material, and other content of the cultural relics. Not all of this information is useful, and most of it is irrelevant and useless. For those useless information, they can be deleted, resulting in 12 main attributes, as shown in Table 1.

**Table 1:** Cultural relics information table.

Serial number	Field name	Data type	Field description	Constraints
1	Name	Int	Unique identifier for cultural relics records	Primary key
2	Subject	Varchar	The type of theme where the cultural relics are located	NO
3	Unearthed site	Varchar	The province where the cultural relics were unearthed	Not null
4	Dynasty to which it belongs	Varchar	The age type of the cultural relics	Not null
5	Function	Varchar	Types of functions of cultural relics	Not null
6	Showcase to which it belongs	Int	Showcase number	NO
7	Museum to which it belongs	Int	Museum number	NO
8	Unearthed time	Date	Time when cultural relics were unearthed	Not null
9	Venue of the exhibition hall	Varchar	Location of cultural relics exhibition	Not null
10	Heritage grade	Varchar	The level of cultural relics stipulated by the state	Not null
11	Material	Varchar	What material are cultural relics made of?	Not null
12	Specification size	Varchar	The size of the cultural relics	Not null

Since this article does not use cultural relic names as identifiers for cultural relics, each cultural relic has its own unique identifier for differentiation. When searching for data, it is found that there are a large number of duplicate cultural relic data, which is an inevitable error in manual data entry because duplicate cultural relic data needs to be deduplicated. In order to avoid duplication of cultural and creative data, the names of cultural relics are used as research objects and the same cultural relics are combined together. After analyzing the data, it is found that some of the data are identical, and such data can be directly deleted. However, there are still some differences in the duplicated data that can be merged for content. By deduplicating and merging duplicate data, cultural relic data is reduced and made more accurate. In addition to data duplication, there is also a problem of inconsistent data formats in the collected cultural relics data. Unifying the data format can better improve the quality of recommendation results [23].

Supergroup algorithm can group and cluster cultural relics, images, texts, and data in museums, automatically dividing them into groups with similar features, which helps to create a cohesive and easy-to-use digital exhibition space. According to the characteristics and requirements of the data, the K-means algorithm is adopted to extract key features from exhibition data, and the extracted features are clustered to obtain object clusters with similar features [24,25]. The K-means algorithm is a complex clustering algorithm and one of the simplest unsupervised learning algorithms for solving clustering problems. This algorithm attempts to divide all sample points into k groups, where each sample point is assigned to its closest cluster [26,27].

The datasets of museums are often large and diverse, covering not only the basic characteristics of cultural relics, but also the historical stories behind them, related events, and rich multimedia materials such as images and videos. In this data context, the K-means algorithm has become a very attractive solution with its simple and intuitive characteristics and the ability to efficiently process large-scale datasets. In particular, it is worth mentioning that this algorithm is not only suitable for processing numerical data, but can also easily handle unstructured data in museums through appropriate preprocessing techniques such as text vectorization and image feature extraction. This wide applicability makes the K-means algorithm particularly excellent when processing complex museum data. Through the application of the K-means algorithm, museums can classify cultural relics according to different characteristics, and conduct targeted thematic displays on digital display platforms. This not only helps the audience to better understand the historical and cultural background of cultural relics, but also provides the audience with more comprehensive and in-depth information services by revealing the potential associations between cultural relics. In summary, the application of the K-means algorithm in the museum's digital display platform has shown significant advantages and practical significance, which has greatly enhanced the audience's tour experience.

Given a sample set $E=\left\{a_{1},a_{2},\cdots ,a_{m}\right\}$, the K-means algorithm divides the cluster obtained from clustering into $X=\left\{X_{1},X_{2},\cdots {,X}_{n}\text{,}\right\}$. The minimum squared error is as follows:

(1) $$H=\sum_{i=1}^{n}\sum_{a=X_{i}}Qa-w_{i}Q_{2}^{2}$$

Among them, $w_{i}=\frac{1}{\left|X_{i}\right|}\sum_{a\in X_{i}}a$ is the mean vector of cluster $X_{i}$.

Assuming that audience x has a residence time before collection y, the degree of audience x’s liking for collection y is expressed as:

(2) $${Interst}_{x,y}=\frac{{time}_{y}}{\max_{i\in y}\left({time}_{i}\right)}$$

Based on the preprocessed cultural relics data, 12 main attribute features of cultural relics are selected, and the correlation degree of cultural relics in Museum A is analyzed. The similarity $sim\left(y_{1},y_{2}\right)$ between collections $y_{1}$ and $y_{2}$ can be expressed as:

(3) $$sim\left(y_{1},y_{2}\right)=a*{sim}_{topic}+b-{sim}_{size}+H+r*{sim}_{office}$$

The location of its excavation varies from dynasty to dynasty. Although cultural relics have different characteristics and attributes, there are still some similarities between them. Therefore, the following similarity formula is used for the study:

(4) $${sim}_{dynasty}=\frac{1}{dist\left({dynasty}_{1},{synasty}_{2}\right)+1}$$

The purpose of adding 1 to the denominator is to prevent it from becoming 0. Even when studying cultural relics from the same period, this formula can be used, and the value range of ${\text{sim}}_{\text{dynasty}}$ is [0,1].

The K-means algorithm is a simple and efficient unsupervised learning algorithm. The basic principle is to divide the data point set into K independent clusters or clusters, and each data point belongs to the cluster represented by the nearest cluster center. In order to allow users to better understand how to adjust the parameters of the K-means algorithm and how these adjustments affect the stability of the results, first of all, a reasonable range of K values can be preliminarily determined by combining domain knowledge and the needs of practical problems. Then, by trying different K values and using evaluation indicators to evaluate the effect of clustering, the best K value is selected. In this process, the “Elbow law” can be used to observe the trend of the clustering effect as the K value increases. The core idea of this law is that after reaching a certain point (that is, the elbow), the increase in clustering effect caused by continuing to increase the K value is significantly weakened. In addition, the maximum number of iterations is another important parameter in the K-means algorithm, which determines the maximum number of iteration rounds for the algorithm to run. If the algorithm has reached the maximum number of iterations before reaching the convergence threshold, then it can be considered to increase the value of max_iter. Generally speaking, for convex data sets, setting a smaller max_iter may be sufficient.

Compared with other clustering methods, such as hierarchical clustering, DBSCAN, and spectral clustering, the adjustment parameters of the K-means algorithm are more intuitive and easy to understand. However, each clustering method has its applicable data sets and problem types, so which method to choose depends on being determined case-by-case. Hierarchical clustering is suitable to reveal the hierarchical structure of the data, while DBSCAN is more suitable to the datasets of clusters with different shapes and sizes.

### 2.2 User demand survey

Through the study of user age and product demand of similar digital museums, it is found that Museum A belongs to a type of museum with strong cultural characteristics, so traditional product research cannot be referred to. This article mainly draws on digital museums with similar characteristics, such as the Palace Museum and the China Intangible Cultural Heritage Digital Museum, and combines many other user research methods to create a reference model. A survey is mainly conducted on 200 users through a questionnaire survey.

In order to select a representative sample, reference is made to Museum A's past visitor data, social media interaction information and user behavior habits, and user groups that meet specific conditions are carefully selected. These conditions involve age group, gender ratio, geographical distribution and frequency of visits. The main research objects are people who are interested in cultural theme museums, especially those who have visited the Palace Museum or the Digital Museum of Chinese Intangible Cultural Heritage. To ensure the reliability of the study, 200 respondents are identified. Data collection is mainly achieved through online questionnaires to gain an in-depth understanding of users' views, expectations and suggestions for improvement on the digital display platform of the Museum A.

Taking into account the voluntary nature of the questionnaire survey, it may make users who are not interested or dissatisfied with the Museum A digital platform choose not to participate. At the same time, respondents may have reservations or give false answers when filling in. In response to these questions, attractive and easy-to-complete questionnaires can be designed to increase users' enthusiasm for participation. At the beginning of the questionnaire, the meaning and purpose of the survey are clarified in order to increase the attention of the respondents. In addition, in addition to questionnaires, interviews and field observations are also supplemented to collect more comprehensive and authentic user feedback. These measures reduce potential biases and ensure the accuracy and representativeness of the data, thereby providing a solid data support for the improvement of the Museum A's digital display platform. Through the collection of online questionnaires, there are 180 valid questionnaires with a 90 % effective rate. The specific information of the surveyed subjects is shown in Table 2.

**Table 2:** Basic information of survey subjects.

Indicator	Classification	Number of people	Percentage (%)
Gender	Male	90	50
	Female	90	50
Age	Under 20 years old	30	16.67
	21–40 years old	80	44.44
	41–60 years old	50	27.78
	Over 61 years old	20	11.11
Education level	Junior high school and below	10	5.56
	High school, college	30	16.67
	Undergraduate	80	44.44
	Master's degree and above	60	33.33
Understanding the situation on the museum's digital platform	Know very well	50	27.78
	Understand	70	38.89
	Do not understand	60	33.33

As shown in Table 2, the research subjects are mainly concentrated between the ages of 21 and 60, accounting for 72.22 %. The number of users aged 61 and above is the lowest, with only 20 people. The user with the highest proportion of education is undergraduate, with 80 people accounting for 44.44 %. The number of users with junior high school and below is relatively small, with only 10 people, and the majority of them are students.

By analyzing the behavior data of extracted users on the museum digital platform, the super swarm algorithm can identify user groups with similar interests or preferences. This helps museums better understand their audience and provide personalized services to them. Digital Museum A is a website that includes culture and education, and it can be seen that the user stickiness of this website is not as high as that of social media or commercial websites. By studying the extracted user information, the visitor characteristics required for Digital Museum A can be better understood, and combined with other data analysis, the user group of Museum A can be generally divided into four categories: ordinary tourists, student groups, experts and scholars, and platform managers.

(1) Ordinary tourists: They seek novelty, focus on leisure and entertainment, and have no fixed tasks and time, with in a state of casual viewing. For this type of visitors, interesting designs can be added to enhance the interest of ordinary tourists, thereby transforming them into information disseminators for Museum A.
(2) Experts, scholars, professionals, and researchers: This group has specific needs and goals, and they are mostly university teachers or researchers from research centers. For the platform, there is a high depth of exploration in terms of information content, integrity, knowledge points, etc. Therefore, special considerations need to be designed for them to strive to convert them into fixed users. Special opinions should be made to adapt to ordinary users.

The main group of ordinary tourists and students on the platform: The number of users accounts for more than 70 % of Museum A's digital display platform. These two types of users use the platform mainly to obtain the information they need, usually basic information and travel tips of Museum A, without the need for in-depth information. Experts and scholars are the second group of users on the platform. They enter the internet to obtain the information they need, and their most concerned content is scientific research and education, accounting for more than 30 % of all users. The user types of A digital museum are shown in Table 3.

**Table 3:** User types of Digital Museum A.

Behavior	Ordinary tourists	Student group	Experts and scholars	Platform manager
Motivation	Get the required information, unconscious browsing	Obtain information based on learning tasks	The need to obtain the required information is clearer	Complete specific work
Search method	Text input is the main, followed by voice input	Text input is mainly	Text input is mainly	Text input is the main, followed by voice input
Client	Mobile phone-based	Computer-based	Computers and mobile phones are equally important	Computer-based
Status	For short-term inquiries, it is difficult to concentrate for a long time	Short-term query, high concentration	Browse for a long time	Depends on the workload
Demand	For pictures of cultural relics, the pursuit of visual stimulation	Interested in cultural relics science	You can look for professional knowledge of museum cultural relics	Mainly to maintain the platform

Through the investigation and analysis of the above research and previous user behavior on similar websites, it can be found that the user group of digital museums is less affected by gender and has a significant correlation with their level of education. From a demand perspective, whether it is ordinary tourists or experts and scholars, their needs are the most active, hoping to quickly find the information they need. In terms of experience, they hope that the interface design is humanized and the operation is “smooth”. The analysis of user needs at different levels of Museum A is shown in Table 4.

**Table 4:** User needs at different levels of Museum A.

Demand	Ordinary tourists	Student group	Experts and scholars	Platform manager
Functional requirements	Provide and be able to quickly find the required information, with clear content and clear framework	Provide and be able to quickly find the required information, with clear content and clear framework	You can quickly find the expertise you need	Easy to operate
Data requirements	The picture is clear and provides basic copy and download functions	The picture is clear and downloaded, the text can be copied	The content is clear and the framework is clear	Rich data sources
Environmental requirements	Fast loading speed and good compatibility	Fast loading speed and good compatibility	Fast loading speed and good compatibility	Low technical threshold and clear procedures
Experience requirements	Smooth operation	Comfortable operation process	The operation process is comfortable and the download speed is fast	Smooth operation
Service requirements	Meet the function of cultural relics interpretation	Meet the function of cultural relics interpretation	Can help in all aspects of the source of cultural relics	Provide services to users

## 3 Construction of knowledge graph of cultural relics in digital museums

The knowledge graph of museums is created, and entities, attributes, and relationships related to museums are modeled [28]. Natural language processing techniques and entity recognition tools are used to extract entities and relationships from data files to assist in the final construction of a knowledge graph. Generally speaking, the construction of a knowledge graph typically includes three models: knowledge extraction, knowledge fusion, and knowledge processing [29,30]. However, due to the large number of cultural relics in museums, different cultural relics have different knowledge attributes and dissemination methods, and the construction of a knowledge graph of museum cultural relics needs to consider more aspects. Therefore, before constructing a knowledge graph of digital cultural relics in museums, the information in the database needs to be processed to unify the data format as much as possible, in order to facilitate the extraction and recognition of museum cultural relics knowledge, and ensure the accuracy and official nature of the knowledge graph.

**Fig. 1:**
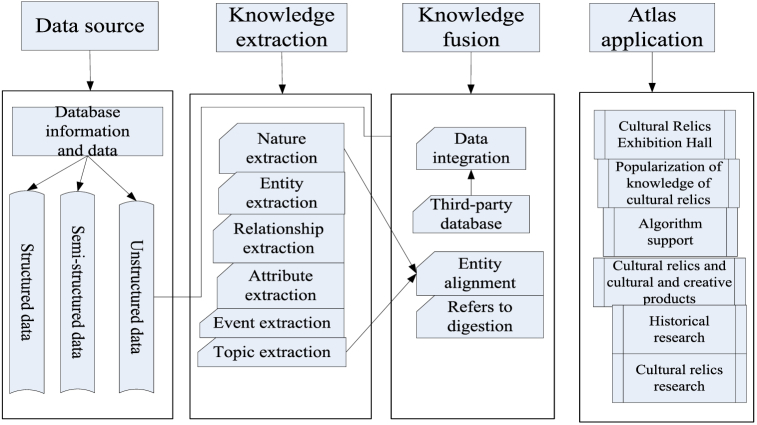
Schematic diagram for constructing a knowledge graph of museum cultural relics.

There is a lack of common standards and formats for museum data, which leads to the construction of knowledge graph and the complexity of interoperability between different museum systems. This lack of standardization seriously hinders the data integration, so the following measures should be taken to promote the standardization and integration of museum data: through the strength of industry associations, government departments or professional institutions, unified data standards and formats should be formulated and popularized to standardize the collection, storage and exchange of museum data. Supergroup algorithm is used to help deal with unstructured data, improve the efficiency of data cleaning and conversion, and reduce the technical difficulty of knowledge graph construction. Museums should strengthen cooperation and jointly promote data standardization and integration. By sharing resources and technical experience, the input cost of data standardization in a single museum can be reduced to promote the progress and development of the whole industry. The schematic diagram for constructing a knowledge graph of museum cultural relics is shown in Fig. 1.

In the past, most museums mainly relied on manual collection to collect basic information on cultural relics. They collect data on cultural relics from various literature, websites, and other museum official websites, in order to build their own museum cultural relics database. However, relying on manual data collection is time-consuming and labor-intensive. Therefore, this article combines network access with expert evidence to improve the reliability of manual data collection. CIDOC-CRM (International Committee for Documentation-Conceptual Reference Model) [31,32] is suitable for the field of cultural heritage literature. After years of continuous improvement, it has 90 entities and 148 genera, covering various aspects of cultural relics. It provides definitions and formal structures to describe the explicit and implicit definitions used in cultural heritage, as well as their relationships, thereby helping researchers, scientists, and platform users from multiple fields to find the specific information they need in the presented databases, and promoting public co construction and sharing of cultural heritage information.

### 3.1 Data processing

Database preprocessing refers to the systematic standardization of the original data sources of the constructed museum cultural relic knowledge graph, solving the problem of database interoperability and laying the foundation for the next development of museum cultural relic knowledge graphs. However, a lot of knowledge about device construction is already included in the previous process, but due to the complexity and diversity of museum cultural relics, there are many problems that require manual intervention. This is mainly because museum collections usually have uniqueness, historical background and complex physical characteristics, which make automatic processing challenging. Museum cultural relics usually have unique cultural, historical and artistic values, and this professional knowledge can only be provided by human experts, because it is difficult for machines to fully understand and explain the historical and cultural background of cultural relics. The physical characteristics of cultural relics, such as material, size, shape and preservation, put forward special requirements for digital processing, and these decision-making and processing processes usually require manual intervention and supervision. This article uses the supergroup algorithm to select a metadata model with clear logic, strong scalability, and the ability to handle data heterogeneity from a semantic level, and maps the database location to a complete metadata model to complete the preprocessing process of the database.

The collected models and data crawled by the network both contain noise, and the data needs to be cleaned. The quality of data cleaning results affects the quality of the final model and the accuracy of museum cultural relics basic data, so the cleaning of cultural relics data is important. If there are some missing cultural relics data scanned during sampling, they should be filled in according to the missing situation and the size of the missing. The missing area is very large and important, so it should be photographed on site. For smaller parts, modeling tools can be used to complete them. There may be some issues with data crawled through the network, such as data consistency, missing important values, and so on. When the same cultural relic has multiple names written in different records, or the purpose of the relic is different, and there is a conflict in the academic community, experts need to provide assistance. The main method for handling missing values in data is to conduct research through manual completion, by removing duplicates and fuzzy data from duplicate fields, and finally, after expert review and approval, they can be stored in the database [33,34].

There are often a lot of missing values and inconsistencies in cultural relics data on the network, such as missing fields, repeated data, format errors and so on. These problems may lead to the decline of data quality and affect the subsequent data analysis and mining. In order to deal with these challenges effectively, before data cleaning, data should be preprocessed, including data deduplication, format conversion, field alignment, etc., to ensure data consistency and comparability. According to the characteristics of cultural relics data, a set of unified cleaning rules is formulated, including dealing with missing values, correcting erroneous data, standardizing data formats, etc., to ensure the quality and accuracy of data. For a large number of data captured by the network, machine learning technology can be used for automatic cleaning. It is necessary to strengthen cooperation and sharing with other museums, research institutions, etc., and jointly formulate data cleaning standards and methods to reduce the cost and improve efficiency of data cleaning in a single institution.

### 3.2 Construction of cultural relics ontology

**Fig. 2:**
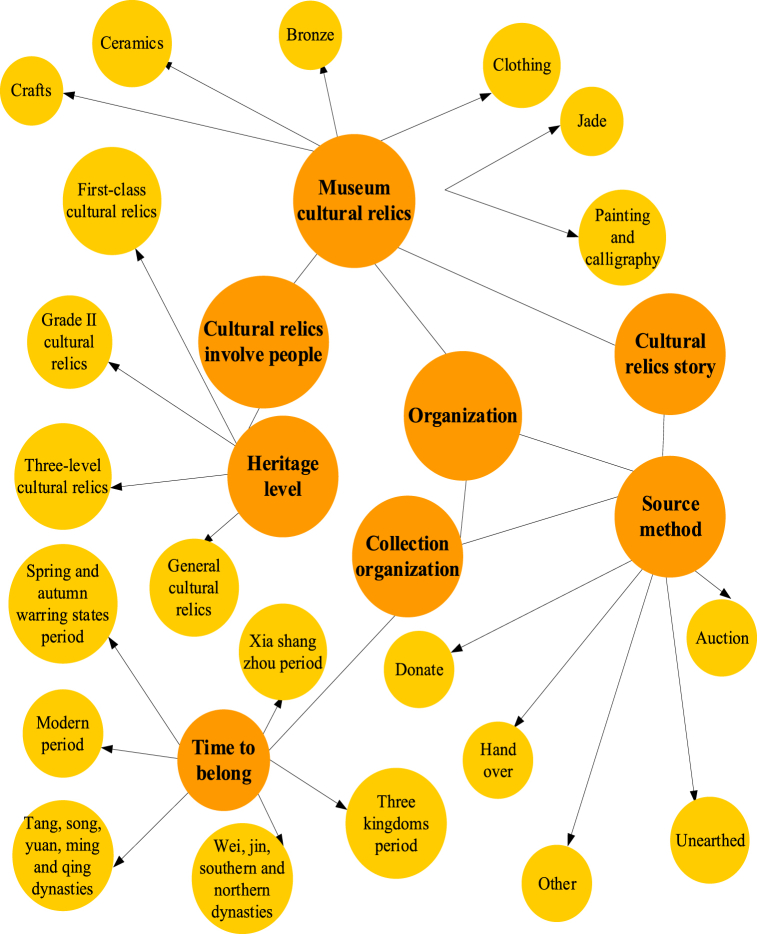
Model of museum cultural relics.

Knowledge graph technology uses ontology concepts to constrain data, typically including definitions of classes, attributes, and relationships [35]. Class represents a description of entity classes that have commonalities. There may be a relationship between the two categories. In the museum cultural relic knowledge graph, the category of “time” is defined, which can be divided into two categories: “excavation time” and “discovery time”, but they are also collectively referred to as the “time” category. When there is a relationship between two classes, it can be described as an object relationship. When one class is defined as “excavation site” and another is defined as “cultural relic”, there is a description of what the “excavation site” of the “cultural relic” is. The model diagram of the museum's cultural relics is shown in Fig. 2.

Artificial entity extraction based on the concept of ontology may indeed introduce subjectivity and bias, which has an impact on the accuracy and reliability of the knowledge graph. This is mainly because artificial entity extraction usually depends on the knowledge and judgment of experts in the field, and this knowledge and judgment may be affected by various factors. In order to reduce the impact of subjectivity and prejudice on the knowledge graph, the following measures can be taken.

1. Standardization and standardization process: A standardized entity extraction process is established to ensure that all participants follow the same rules and guidelines. This can reduce the differences between different experts and improve the consistency and reliability of the extraction results.
2. Multi-expert verification: Experts from multiple fields are aplied to participate in the entity extraction process, and the extraction results are cross-validated to help correct the errors and biases of a single expert and improve the accuracy of the extraction results.
3. Use of automated tools and algorithms: Supergroup algorithms are combined for entity extraction to reduce dependence on manual intervention and automatically extract entities from large-scale data.

### 3.3 Knowledge extraction

**Fig. 3:**
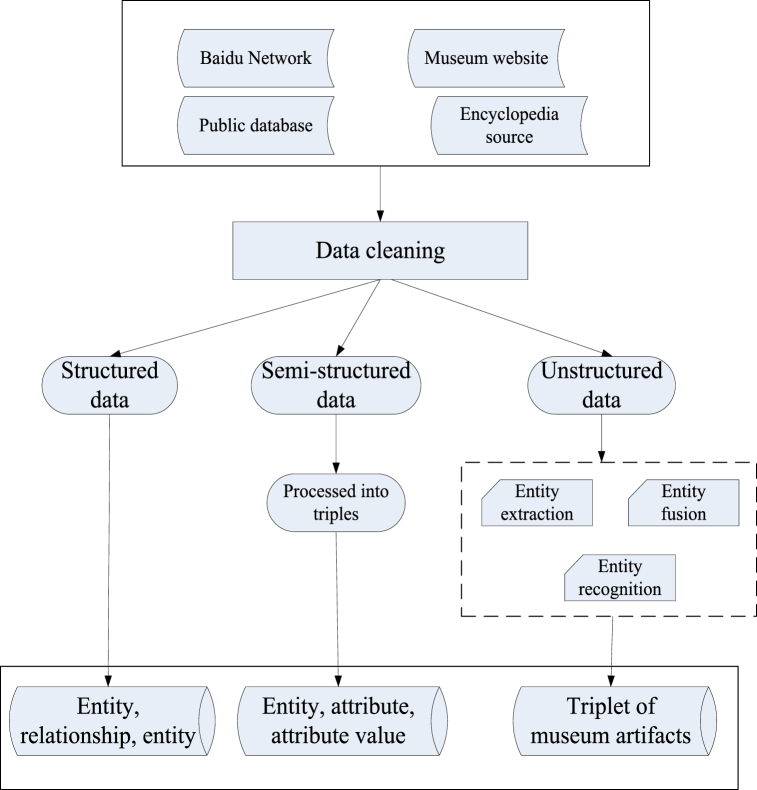
Schematic diagram of the overall knowledge extraction module.

There are two types of data sources for knowledge extraction of museum cultural relics: semi-structured data sources and unstructured data sources [36,37]. By using the K-means algorithm, the seed keywords filtered from the database and the semi-structured data obtained from the network platform can be transformed into triplet information after certain processing. The sentence information of relevant cultural relics crawled from the network platform through the K-means algorithm, as well as some cultural relic data filtered out from the metadatabase, are all unstructured data. For non essential cultural relic information, it is necessary to obtain triplets through named entity recognition and relationship extraction. Among them, named entity recognition and relationship extraction are mainly implemented using the K-means algorithm. The schematic diagram of the overall knowledge extraction module is shown in Fig. 3.

Considering the low level of digitalization of cultural relics records in Museum A, the large and extensive content of the records, and the lack of relevant training datasets, the automatic data extraction effect is not satisfactory. The low digitalization degree of museum heritage records mainly faces the challenges of technology implementation and update, data quality and integrity challenges, security and privacy challenges, and training and human resources challenges. This article designs tools to automatically identify and extract entities and attributes in heritage records, which can partly reduce manual intervention, improve processing efficiency, and reduce the impact of subjectivity on results. Unified data extraction standards and procedures are developed to ensure that different personnel follow the same norms and standards in data extraction. This helps to reduce subjectivity and improve data consistency and accuracy. In the data extraction guide, Excel spreadsheets are created, labeled with subcategories and grouped to store items, and entity attributes are displayed sequentially. The identification number related information is input into the section based on ontology object attributes to ensure the accuracy of the established relationships between entities. More than 600 entities are ultimately extracted, and over 2000 relationships between entities are established.

### 3.4 Knowledge processing and integration

**Fig. 4:**
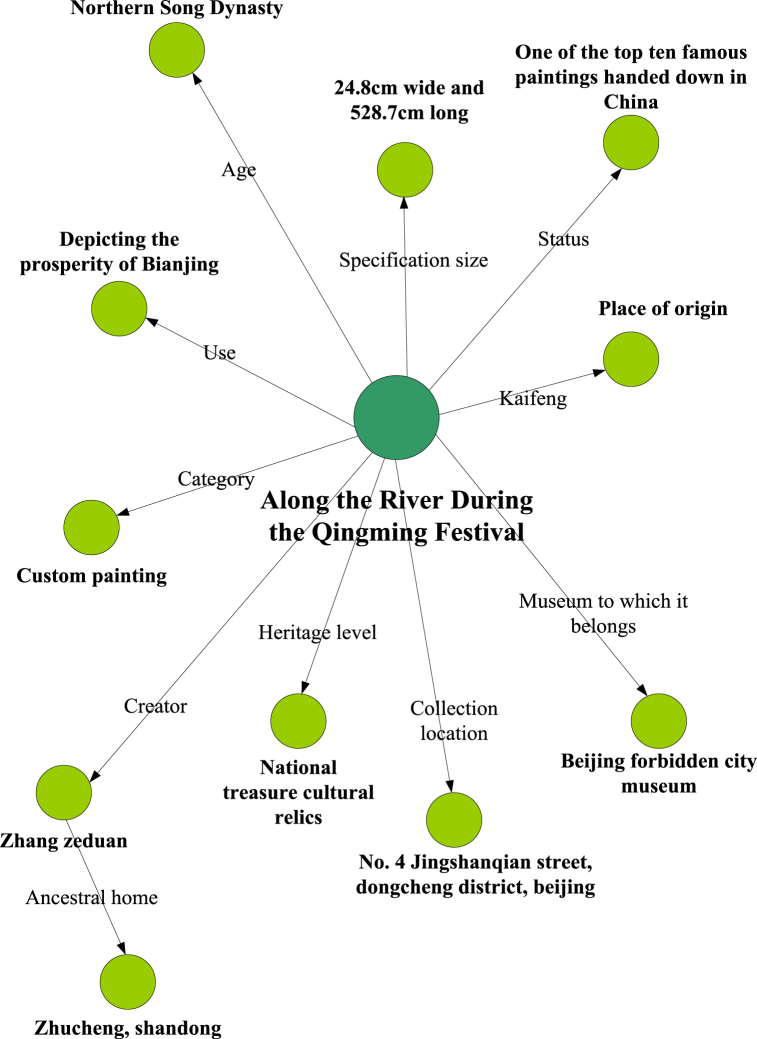
Knowledge graph of along the River During the Qingming Festival.

After completing knowledge extraction, knowledge fusion should be carried out to ensure the accuracy of knowledge graph construction. The main problem of this study is the problem of the same organization describing the same entity differently in different data. Different data sources may adopt different data formats, structures, and standards, complicating data integration. There may also be quality differences, and the integration of data from different sources may involve the processing of sensitive information and privacy data, requiring appropriate security measures and privacy protection schemes to ensure the security and privacy of the data. To address this issue, the characteristics of each entity attribute are compared. Data combination is adopted to store cultural relic knowledge, and knowledge fusion is the fusion of information from different sources. After knowledge extraction is completed, it contains a lot of relevant information. In order to ensure the integrity of the knowledge, it is necessary to remove interfering information. For example, in “the Along the River During the Qingming Festival is currently stored at No. 4 Jingshanqian Street, Dongcheng District, Beijing”, the strings “Along the River During the Qingming Festival”, “Beijing City”, “Dongcheng District”, and “Jingshanqian Street” should be mapped to their respective entities. In many cases, the same organization has different names, or the same name has different locations, so disambiguation and co referential resolution are required during the registration process. Secondly, knowledge sharing is needed to make data more interesting and comprehensive. The solution is to obtain knowledge from one's own knowledge base as input and supplement entity attribute values. The knowledge graph of the Along the River During the Qingming Festival of cultural relics is shown in Fig. 4.

### 3.5 Knowledge graph maintenance

The maintenance of knowledge graph is a key process to ensure the accuracy and relevance of its data.Through the establishment of data quality standards and quality control mechanisms, data is checked and cleaned regularly, and regularly updated to reflect new information and facts. Entity links and relationships need to be checked and updated regularly to ensure that they are still valid and accurate. Users are encouraged to provide feedback, report bugs or provide new information. With the advancement of technology and the emergence of new methods, it is necessary to continuously optimize and improve the maintenance strategy of the knowledge graph. This may involve the introduction of new technologies, methods, or tools to improve data quality and accuracy.

Building and maintaining the knowledge graph of museum cultural relics really needs a lot of resources, including time, professional knowledge and computing power. These resource intensities may indeed limit the adoption of some small museums, especially those institutions with limited resources. The following are some suggestions to help small museums use limited resources to build and maintain knowledge graphs more effectively.

1) Cooperation and sharing: Small museums can establish cooperative relations with other small museums, academic institutions or large museums to jointly build and maintain knowledge graphs. Through cooperation, resources and expertise can be shared to reduce costs and improve efficiency. In addition, participating in the shared knowledge graph project can also enable small museums to benefit from the experience and best practices of other institutions.
2) Priority setting and phased implementation: Small museums should set the priority of building and maintaining knowledge graphs according to their own resources and needs, and implement them in stages. Some cultural relics or themes can be first selected to construct knowledge graphs, and gradually expanded to other fields. In this way, a complete and reliable knowledge graph can be gradually established with limited resources.
3) Seeking external support and funds: Small museums can actively seek external support and funds to promote the construction and maintenance of knowledge graphs.

## 4 Experiment on the knowledge graph of digital cultural relics display in museums

**Fig. 5:**
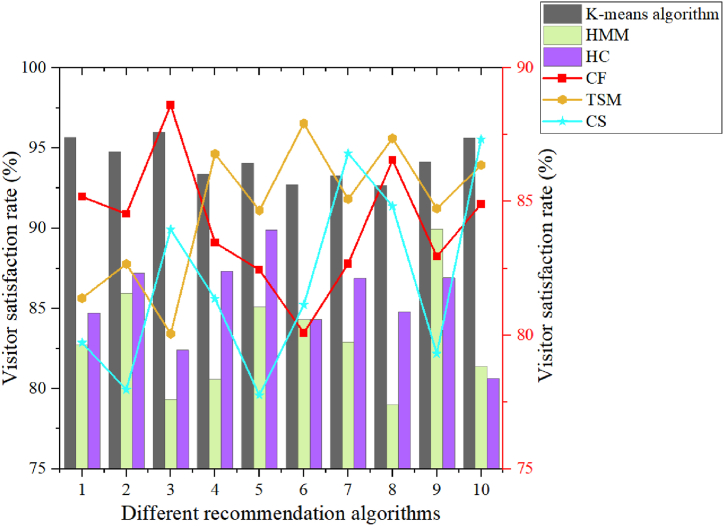
Comparison of satisfaction rates of visitors with cultural relic recommendation algorithms.

The K-means algorithm in the super swarm algorithm was used to investigate the needs of users for visiting cultural relics, in order to recommend suitable museums and cultural relics for visitors based on their needs, and achieve personalized visits. This article mainly conducted research on Museum A. The museum had 10000 exhibits on display and basic information about 180 visitors. These 180 viewers were recommended for cultural relics, mainly to study the satisfaction rate of the recommendation results. In the experiment, cross validation was adopted, and 10 experiments were conducted. The average of each experiment result was taken as the recommendation result output, and the output result was compared with the results obtained based on Collaborative Filtering (CF) algorithm, Hidden Markov Model (HMM), Time Series Modeling (TSM) algorithm, Hierarchical Clustering (HC), and Cosine Similarity (CS). To ensure objective comparison with algorithms CF, HMM, TSM, HC, CS, the same or similar datasets were used to ensure the consistency of data distribution, scale and characteristics. The appropriate parameter settings were carefully selected for each algorithm, ensuring that they were all run under the same conditions. In order to comprehensively evaluate the performance of the algorithm, many evaluation indexes were adopted, which can reveal the advantages and applicability of the algorithm from different angles. For different types of algorithms, targeted evaluation indicators were selected for comparison. Furthermore, experiments were performed on multiple datasets and repeated several times to remove interference from random factors. By comparing the results of different datasets and multiple experiments, the stability and robustness of the algorithm can be judged more precisely. The specific comparison results are shown in Fig. 5.

In Fig. 5, the x-axis represents different recommendation algorithms; the left y-axis represents the satisfaction rate of visitors under the K-means algorithm, HMM, and HC algorithm, while the right y-axis represents the satisfaction rate of visitors under the CF, TSM, and CS algorithms. Through 10 experiments, it can be found that visitors were much more satisfied with the algorithm studied in this article than other algorithms. This also indicates that using the K-means algorithm to recommend cultural relics to visitors is more in line with their needs and can better achieve personalized cultural relic recommendations. Among them, the visitor satisfaction rate based on K-means algorithm was above 92.68 %, while the visitor satisfaction rates based on CF, HMM, TSM, HC, and CS algorithms were below 88.62 %, 89.97 %, 87.94 %, 89.94 %, and 87.34 %, respectively. In the third experiment, the satisfaction rate of the algorithm studied in this article was 96.02 %, which was 7.41 %, 16.67 %, 15.96 %, 13.59 %, and 12.06 % higher than the satisfaction rates based on CF, HMM, TSM, HC, and CS algorithms, respectively. Meanwhile, the visitor satisfaction rate of the algorithm studied in this article was 94.25 % in 10 experiments, which were 10.1 %, 11.11 %, 9.54 %, 8.73 %, and 12.23 % higher than those based on CF, HMM, TSM, HC, and CS algorithms, respectively. Using the K-means algorithm in conjunction with supervised information may improve clustering accuracy and thus indirectly increase visitor satisfaction. The following main concerns and potential issues also need to be considered: data quality questions, parameter selection questions, and interpretability questions. Only by fully considering these questions and taking corresponding measures can the supergroup algorithm achieve good results in practical applications.

The satisfaction used is investigated in Fig. 5, and in order to study the significant relationship between different algorithms and user satisfaction, a statistical analysis was performed. The results obtained are shown in Table 5.

**Table 5:** User satisfaction study under different algorithms.

Algorithm	Satisfaction rate (%)	Satisfaction standard deviation	P value
K-means	94.25	2.5	0.001
CF	84.15	3.1	0.082
HMM	83.14	2.8	0.126
TSM	84.71	3.3	0.043
HC	85.52	2.6	0.038
CS	82.02	3.4	0.161

Through statistical analysis, the results shown in Table 5 are obtained. First of all, the K-means algorithm performed the most outstanding in terms of user satisfaction, with a satisfaction rate of 94.25 % and a standard deviation of 2.5 in satisfaction, which showed the consistency and high level of user satisfaction. In addition, the P-value of the K-means algorithm was 0.001, which was much lower than the significance level of 0.05, which means that the K-means algorithm has significant advantages over other algorithms in improving user satisfaction. On the other hand, although the CF, HMM, TSM, HC and CS algorithms performed well in user satisfaction, the satisfaction was low compared to the K-means algorithm. Among them, the P-values of TSM and HC were 0.043 and 0.038, respectively, both showing significant differences in user satisfaction with the K-means algorithm. These data show that when building a museum digital display platform, the use of K-means algorithm can significantly improve user satisfaction. Compared with other algorithms, the K-means algorithm is more accurate in clustering user preferences and interests, thereby providing users with more personalized and accurate display content, thereby improving user satisfaction.

The first task of creating a knowledge graph for digital display of museum cultural relics is to construct a dataset, which mainly consists of two parts: a training set and a testing set. The main focus is to study the Chinese cultural relics in Museum A mentioned earlier, including data on seven categories of cultural relics: porcelain, clothing, calligraphy and painting works, gold and silver cultural relics, musical instruments, pottery, sculptures, etc. A total of 2200 pieces were studied, including structural information such as cultural relic numbers, names, categories, and ages, totaling 2600 pieces. Using web scraping technology, data on 7 categories of cultural relics extracted from encyclopedia websites was collected, with 2400 items collected, totaling 5000 items. These data were processed to remove duplicate data and obtain the final experimental data. The specific data is shown in Table 6.

**Table 6:** Number of entity categories in the training and testing sets.

Serial number	Entity category	Number of training sets	Testing set data	Total
1	Porcelain	688	172	860
2	Clothing	400	100	500
3	Calligraphy and painting works	336	84	420
4	Gold and silverware	864	216	1080
5	Musical instrument	528	132	660
6	Pottery	640	160	800
7	Sculpture	544	136	680
Total		4000	1000	5000

In order to study the performance of the museum cultural relic display knowledge graph constructed based on the K-means algorithm, it was tested using three evaluation indicators: accuracy, recall, and F1 value. The test results were compared with models constructed based on Named Entity Recognition (NER), Recurrent Neural Network (RNN), Convolutional Neural Network (CNN), Graph Neural Network (GNN), String Matching (SM), Random Forest (RF), and Naive Bayes model (NBM). Meanwhile, in order to achieve fairness in the final results, all models used the same training set data, and all model parameters used for training remain consistent. During the training process, the results of each training session were recorded. The specific research results are shown in Table 7.

**Table 7:** Comparison of experimental results on the performance of knowledge graphs for displaying cultural relics in different museums.

Model	Accuracy	Recall rate	F1 value
K-means algorithm	90.12 %	84.69 %	82.23 %
NER	82.35 %	73.83 %	62.33 %
RNN	77.21 %	72.48 %	65.69 %
CNN	63.59 %	60.36 %	50.14 %
GNN	80.09 %	74.82 %	70.29 %
SM	78.68 %	72.46 %	65.89 %
RF	69.52 %	66.18 %	62.68 %
NBM	73.18 %	69.33 %	58.68 %

As shown in Tables 7 and it can be observed that the knowledge graph studied in this article had higher accuracy, recall, and F1 value than other models. The performance of the museum cultural relic knowledge graph constructed in this article was even better. Based on the K-means algorithm, a knowledge graph was constructed with an accuracy of 90.12 %, which was 7.77 %, 12.91 %, 26.53 %, 10.03 %, 11.44 %, 20.6 %, and 16.94 % higher than the models constructed by NER, RNN, CNN, GNN, SM, RF, and NBM, respectively. The recall rate of the knowledge graph studied in this article was 84.69 %, which was 10.86 %, 12.21 %, 24.33 %, 9.87 %, 12.23 %, 18.51 %, and 15.36 % higher than the accuracy of the models constructed by NER, RNN, CNN, GNN, SM, RF, and NBM, respectively. The F1 value of the knowledge graph studied in this article was 82.23 %, which was 19.9 %, 16.54 %, 32.09 %, 11.94 %, 16.34 %, 19.55 %, and 23.55 % higher than the accuracy of the models constructed by NER, RNN, CNN, GNN, SM, RF, and NBM, respectively. In summary, the museum cultural relic knowledge graph constructed based on the K-means algorithm performed better.

**Fig. 6:**
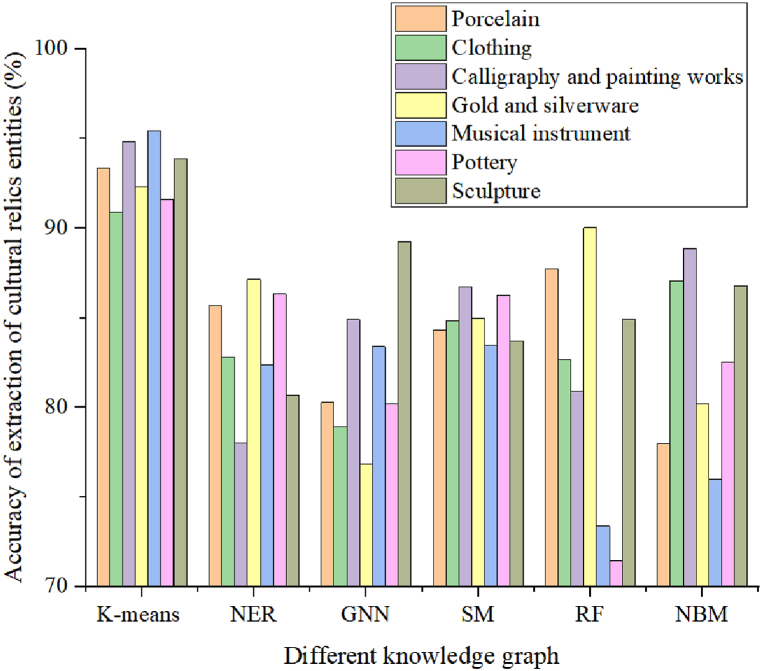
Comparison of accuracy of knowledge extraction from museum cultural relics using different knowledge graphs.

Seven categories of cultural relics were extracted from Table 5, and their category knowledge was extracted and identified. It was found that the museum cultural relic knowledge graph constructed based on the K-means algorithm had a higher accuracy in extracting and identifying the knowledge of these cultural relics. The experimental results were compared with knowledge graphs constructed based on NER, GNN, SM, RF, and NBM algorithms. The specific comparison results are shown in Fig. 6.

In Fig. 6, the x-axis represents the museum cultural relic knowledge graph constructed by different methods, and the y-axis represents the accuracy of museum cultural relic knowledge extraction. As shown in Figs. 6 and 7 cultural relics were extracted, and the knowledge graph constructed in this article had a higher accuracy in extracting knowledge data from these 7 cultural relics compared to the knowledge graphs constructed by other methods. The accuracy of knowledge extraction for cultural relics such as calligraphy and painting works was 94.81 %, which was 16.79 %, 9.89 %, 8.09 %, 13.89 %, and 5.96 % higher than the accuracy of knowledge graph extraction constructed by NER, GNN, SM, RF, and NBM algorithms, respectively. The accuracy of extracting knowledge from cultural relics such as musical instruments was 95.45 %, which was 13.06 %, 12.06 %, 12.01 %, 22.08 %, and 19.49 % higher than the knowledge graph constructed by NER, GNN, SM, RF, and NBM algorithms, respectively. This article mainly extracted knowledge data from 7 cultural relics, and constructed a knowledge graph based on K-means algorithm. The average accuracy of entity knowledge data extraction for these 7 cultural relics was 93.19 %, which was 9.9 %, 11.22 %, 8.3 %, 11.61 %, and 10.43 % higher than the average accuracy of NER, GNN, SM, RF, and NBM algorithms, respectively.

Specific algorithms such as CF, HMM, TSM, and HC were selected as the baseline, mainly because of their respective representativeness. As a widely used personalized recommendation algorithm, CF can recommend similar items or services based on the user's historical behavior. HMM is a statistical model describing Markov processes with implicit unknown parameters and is well suited for sequence data analysis. TSM focuses on the regularity of data change over time and is often used in prediction and pattern recognition. While HC, as a clustering method, reveals the hierarchical structure of the data by dividing the data layer by layer. These algorithms represent different technical directions and applicable scenarios, and can be used as baselines to comprehensively evaluate the performance of K-means algorithm in various aspects.

With the continuous growth of museum collections, scalability has become a crucial issue in the construction of knowledge graphs. The method of manual preprocessing and extraction is no longer efficient in the face of a large number of data, and may even be impossible to achieve. Therefore, it is necessary to consider adopting more scalable automatic and semi-automatic methods to meet this challenge. Using natural language processing (NLP) and computer vision technology, entities can be automatically identified and extracted from texts and images. These technologies can be applied to the description, labels and images of museum collections, so as to extract entity information quickly and accurately. For a large museum, its collection data may be massive. Therefore, large-scale data processing tools, such as distributed computing framework or database management system, are needed to process and analyze these data. These tools can efficiently process large amounts of data and provide scalable storage and computing power. In order to promote the integration and sharing between different data sources, it is necessary to adopt standardized data models and formats to construct knowledge graphs. This can ensure the consistency and interoperability of data and simplify the process of data processing and analysis.

Through clustering, the K-means algorithm groups data or cultural artifacts that are comparable. The digital display platform at the museum offers an easy user experience in part because of this clever grouping technique. In addition to being quite easy to use and apply, the K-means method has a high execution efficiency when applied to large-scale data sets. The K-means algorithm's flexibility and intuitiveness enable it to extract hidden patterns in museum data, despite the potential diversity and complexity of data. Direct and understandable information is more appealing to users of the museum's digital display platform. By using clustering, the K-means algorithm provides a natural grouping that makes it easier for people to comprehend and locate the content they are interested in.

## 5 Conclusions

With the increase in the number of cultural relics in museums, the scale of their information resources is also constantly expanding, which also leads to the expansion of the scale of digital cultural relic data. The increasing demand for digital exhibition platforms in museums poses new demands for the management of knowledge in the field of cultural relics. Knowledge graphs can reveal deep connections between cultural relics by presenting knowledge and describing entity relationships. The knowledge base of cultural relics was constructed to realize the effective sharing of cultural relics resources, providing technical support for the construction of digital museums. This article conducted research on the ontology and knowledge extraction of cultural relics based on general knowledge graph technology and supergroup algorithms, and achieved certain research results. The K-means algorithm was used to mine large and complex data in museum cultural relics, providing personalized and accurate recommendations for cultural relics and exhibits to visitors, helping museums better understand their needs and optimize exhibition layout and content design. Secondly, the use of knowledge graphs on museum digital display platforms was also studied. By constructing a knowledge graph of museum cultural relics, various types of cultural relics knowledge within the museum can be correlated and integrated, forming a rich semantic network.

## Data availability statement

All the data used to support the findings of the study are included within the work.

## CRediT authorship contribution statement

**Liping Su:** Writing – review & editing, Writing – original draft, Resources, Methodology, Data curation, Conceptualization. **Hongli Liu:** Formal analysis, Data curation. **Wenru Zhao:** Writing – review & editing, Writing – original draft, Software, Data curation, Conceptualization.

## Declaration of competing interest

The authors declare that they have no known competing financial interests or personal relationships that could have appeared to influence the work reported in this paper.
